# Socio-economic differences in self-reported insomnia and stress in Finland from 1979 to 2002: a population-based repeated cross-sectional survey

**DOI:** 10.1186/1471-2458-12-650

**Published:** 2012-08-13

**Authors:** Kirsi M Talala, Tuija P Martelin, Ari H Haukkala, Tommi T Härkänen, Ritva S Prättälä

**Affiliations:** 1 Department of Health, Functional Capacity and Welfare, National Institute for Health and Welfare (THL), Helsinki, Finland; 2 Department of Social Research, University of Helsinki, Helsinki, Finland; 3 Department of Lifestyle and Participation, National Institute for Health and Welfare (THL), Helsinki, Finland

**Keywords:** Self-reported insomnia, Self-reported stress, Socio-economic differences, Repeated cross-sectional survey, Time trend

## Abstract

**Background:**

Over the decades, global public health efforts have sought to reduce socio-economic health differences, including differences in mental health. Only a few studies have examined changes in socio-economic differences in psychological symptoms over time. The aim of this study was to assess trends in socio-economic differences in self-reported insomnia and stress over a 24-year time period in Finland.

**Methods:**

The data source is a repeated cross-sectional survey “Health Behaviour and Health among the Finnish Adult Population” (AVTK), from the years 1979 to 2002, divided into five study periods. Indicators for socio-economic status included employment status from the survey, and educational level and household income from the Statistics Finland register data. We studied the age group of 25–64 years (N = 70115; average annual response rate 75%). Outcome measures were single questions of self-reported insomnia and stress.

**Results:**

The overall prevalence of insomnia was 18-19% and that of stress 16-19%. Compared to the first study period, 1979–1982, the prevalence of stress increased until study period 1993–1997. The prevalence of insomnia increased during the last study period, 1998–2002. Respondents who were unemployed or had retired early reported more insomnia and stress over time among both men and women. Lower education was associated with more insomnia especially among men; and conversely, with less stress among both sexes. Compared to the highest household income level, those in the intermediate levels of income had less stress whereas those in the lowest income levels had more stress among both sexes. Income level differences in insomnia were less consistent. In general, socio-economic differences in self-reported insomnia and stress fluctuated some, but did not change substantially over the study period 1979–2002.

**Conclusions:**

Self-reported insomnia and stress were more common during later study periods. The socio-economic differences in insomnia and stress have remained fairly stable over a 24-year time period. However, some of the associations in socio-economic differences were curvilinear and converse. Future studies are needed to explore the complex socio-economic gradients, especially in stress.

## Background

The aim of WHO’s ‘Global strategy for health for all by the year 2000’ was to reduce socio-economic health differences, including those in mental health, and this strategy was also launched in Finland in 1985. Various health indicators show improvement in the health of the population, but socio-economic differences in general health have remained stable or even widened in Finland [1] and other western countries [2-4]. Few studies that have been conducted on psychological symptoms, which are mainly measured as depressive symptoms, also show similar trends [5,6]. Our previous study on trends in socio-economic differences in self-reported depression demonstrated that educational, employment status and household income differences in self-reported depression remained stable during the study period 1979–2002 [6]. However, changes in socio-economic differences in other psychological symptoms have not been widely studied.

Non-specific psychological symptoms, such as self-reported insomnia and stress, are commonly used to monitor the subjective dimension of mental well-being at the general population level [7]. However, measures and definitions of insomnia and stress vary substantially. Generally, insomnia has been defined as difficulty falling asleep or staying asleep despite adequate opportunity for sleep [8]. Insomnia is known to have a major negative impact at both the individual and societal level, including daytime functioning, loss of productivity, absenteeism from work, work accidents [9] and subsequent disability retirement [10]. Stress in turn can be defined as a psychological and physiological response to a situation that threatens or challenges us and requires us to make some kind of adjustment. People perceive and are affected by stress in different ways; in some cases it may be considered to result in good outcomes, while in other cases it can lead to negative outcomes [11,12]. Both insomnia and stress are known to be associated with lower quality of life, morbidity and mortality [10,13-18]. Moreover, there is evidence of a social gradient in both sleeping problems [19-21] and stress [13,22]. For example, sleeping problems have been proposed to be a mechanism through which low SES is linked to poor health [19]. In addition, low SES is correlated with exposure to stressful environments and conditions, such as noise, crime, hazards or privation and poor access to resources, that may contribute to chronic stress [22]. Low basic education and low socio-economic status were found to carry a risk for chronic work-related stress (burnout) among working women but not among men in a Finnish Health 2000 Study [23]. Work-related stress has demonstrated less consistent results with socio-demographic factors than organisational factors [24,25].

Insomnia appears to be highly common in the general population. In a review of epidemiological studies, the prevalence of insomnia symptoms without restrictive criteria (based on “yes-no” answers) was between 30-48% [26]. Overall prevalence of insomnia symptoms in the Finnish population was found to be 37.6% [27]. A comparative review and re-analysis of various survey data in Finland indicated an increase in insomnia-related symptoms during 1995–2005 [28]. Furthermore, in a Swedish population study of women, the prevalence of sleeping problems increased [29] during the 36 years of observation.

Most of the studies regarding stress have focused on the working population and on work-related chronic stress, i.e. burnout [23,24]. Fewer studies have examined the prevalence of self-reported stress in the general population. One estimate is provided by the 1985 National Health Interview Survey in the U.S; the percentage of respondents who experienced “a lot” of stress was 23% among women and 18% among men [11]. In Sweden the proportion of those reporting psychological stress increased between the years 1985 to 1995 amongst women aged 25–34 whereas only little variation was found in men [30]. No study has been conducted on the prevalence of self-reported stress over time in Finland.

To our knowledge, only a few surveys have explored long-term cross-sectional trends in psychological symptoms in a representative general population. In this study we will describe the overall repeated cross-sectional 24-year prevalence and socio-economic differences in self-reported insomnia and stress over the period from 1979 to 2002 among men and women in Finland. The aim of this study is to clarify the following research questions: (1) has the prevalence of self-reported insomnia and stress changed over the study period 1979–2002 and (2) have the educational, employment status and household income level differences in self-reported insomnia and stress changed over the study period 1979-2002?

## Methods

The basic data source was the nationwide repeated cross-sectional survey “Health Behaviour and Health among the Finnish Adult Population”, which has been conducted since 1978 by the National Institute for Health and Welfare (formerly the National Public Health Institute) [31]. The annual questionnaire is mailed to a random sample of 5,000 Finns aged 15–64 years. The sampling was selected using simple random sample conducted by The Finnish Population Information System which is a computerized national register that contains basic on-line information about all Finnish citizens residing permanently in Finland. For this study, data were restricted to 25-64-year-old respondents, and did not include respondents under 25 years, whose socio-economic status is less stable. The total period of time covered in this study was 1979–2002. Survey years were divided into five periods: 1979–82, 1983–87, 1988–92, 1993–97 and 1998–2002. The survey year 1985 was excluded because personal identification codes were missing for that year. The survey data were completed with register data on educational level and household income from Statistics Finland based on the personal identification codes issued to all Finnish citizens living permanently in Finland. After excluding persons with missing data in insomnia and stress variables (N = 1175, 1.6%), the data comprised a total of 70,115 persons (average annual response rate 75%), of whom 33,493 were men (average response rate 71%) and 36,622 women (average response rate 79%). The Institutional Review Board of the National Institute for Health and Welfare (THL) (IRB 00007085, FWA 00014588) has reviewed and supported our research plan.

### Self-reported insomnia and stress

In this study, self-reported insomnia and stress are thought to measure subjective dimensions of psychological well-being at the general population level [7]. Both self-reported insomnia and stress were measured by a single question. For insomnia, the respondents were asked about 14 health problems or symptoms, among them ‘insomnia’, with the following question: “Have you had any of the following symptoms or health problems during past 30 days?” (Yes, if so).

Stress was addressed in a separate four-point scale question: “Have you had symptoms of tension or been under great stress or considerable strain during the past 30 days?” (1 = my life is nearly unbearable (2.5%), 2 = more than people in general (15%), 3 = somewhat but not usually so (60%), 4 = not at all (23%)). Those reporting stress ‘more than people in general’ or ‘my life is nearly unbearable’ were classified as having stress. Pearson’s correlations (p < 0.01) between insomnia and stress were r = 0.29 in males and r = 0.26 in females. We also conducted some additional analyses for ‘my life is nearly unbearable’ -category alone (2.5%) as referring to extremely high stress.

### Employment status

Employment status was queried with the following question: “What kind of work do you do most of the year?” Three occupational categories were given in the questionnaire; ‘agricultural work’, ‘industrial work’, and ‘office work and service’. As being crude measures of occupation, all those categories were grouped together in the ‘employed’ category. Additional categories were ‘unemployed’, ‘student’ (>24 years), ‘retired’ and ‘housewife/house husband’. The results were not reported for the house husband category due to the small number of respondents in this category. The official age of retirement for most occupations in Finland is 65, meaning that in our data, which included working-aged respondents under 65 years, all the retired respondents had taken early retirement (these comprise early old-age pensioners (62–64 years old), part-time pensioners, disability pensioners and unemployment pensioners (60–64 years old)).

### Register-based data on education and household income

The register data for education and income were linked individually from the 1980 statistics for the survey years 1979–1983, from the 1985 statistics for the survey years 1984–1986 and annually from 1987 until 2000. For the survey years 2001–2002, we used the socio-economic data from the year 2000. Educational levels were divided according to the Register of Educational Qualifications and Degrees, which follows the principles and categories of the revised UNESCO International Standard Classification of Education 1997 (ISCED 1997). The lowest level of education included nothing other than lower secondary education or an unknown educational level, the intermediate level included upper secondary or post-secondary non-tertiary education and the highest level included tertiary education.

We chose household income as the indicator for income because compared to individual income it has been shown to be more strongly and consistently associated with health [32]. Income for a household was calculated as annual taxable total gross income without transfer payments. This figure was divided by the consumption unit of the OECD equivalence scale, where the first adult in the household was weighted as 1.0, other adults as 0.7 and underage children as 0.5 [33]. Household income per consumption unit was further divided into quintiles by every study year in order to ensure the comparability of the variable over time.

The general description of the data is presented in Table 1. Among both sexes the proportion of unemployed respondents increased. Furthermore, the proportion accounted for by the higher educated compared to the lower educated increased after the first study period, 1979–1982.

**Table 1:** Description of the data

	**Study periods**										**Total period**	
	**1979–1982**		1983–1987*		**1988–1992**		**1993–1997**		**1998–2002**		**1979–2002**	
	**%**	**N**	**%**	**N**	**%**	**N**	**%**	**N**	**%**	**N**	**%**	**N**
**Men**												
Insomnia	17	1267	16	950	17	1209	18	1193	20	1289	18	5908
Stress	16	1215	17	1023	19	1325	20	1326	20	1298	19	6187
Age												
25-34	32	2378	30	1760	27	1941	24	1589	21	1329	27	8997
35-44	27	2043	30	1752	30	2148	27	1774	26	1667	28	9384
45-54	23	1723	22	1261	23	1645	28	1843	30	1920	25	8392
55-64	18	1314	19	1102	20	1420	21	1405	23	1479	20	6720
Educational level												
Highest	20	1451	22	1297	25	1790	28	1827	33	2071	25	8436
Intermediate	26	1936	31	1784	35	2500	39	2592	41	2596	34	11408
Lowest	54	4033	47	2744	40	2840	33	2173	27	1712	41	13502
Employment status												
Employed	86	6284	85	4951	82	5870	73	4714	78	4738	81	26557
Student	2	109	1	81	2	155	2	139	2	142	2	626
Retired	10	728	11	655	12	881	13	830	12	739	12	3833
Unemployed	2	174	3	150	3	228	12	756	8	481	5	1789
Total N		7458		5875		7154		6611		6395		33493
						
	**1979–1982**		1983–1987*		**1988–1992**		**1993–1997**		**1998–2002**		**1979–2002**	
	**%**	**N**	**%**	**N**	**%**	**N**	**%**	**N**	**%**	**N**	**%**	**N**
**Women**												
Insomnia	19	1292	17	1171	17	1372	19	1408	21	1583	19	6826
Stress	12	830	14	925	15	1198	19	1405	18	1356	16	5714
Age												
25-34	30	2025	28	1883	26	2116	25	1889	22	1649	26	9562
35-44	23	1569	27	1834	30	2434	28	2118	27	1985	27	9940
45-54	22	1487	22	1457	23	1881	28	2113	30	2194	25	9132
55-64	26	1766	23	1552	20	1599	20	1501	21	1570	22	7988
Educational level												
Highest	16	1109	22	1467	26	2079	33	2470	37	2759	27	9884
Intermediate	26	1756	31	2069	35	2778	37	2836	39	2870	34	12309
Lowest	58	3966	47	3146	39	3164	30	2302	24	1756	40	14334
Employment status												
Employed	69	4626	74	4942	75	5993	69	5159	73	5193	72	25913
Student	1	84	2	111	3	218	3	253	3	223	3	889
Housewife	16	1098	12	773	9	698	8	597	6	444	10	3610
Retired	12	775	11	718	11	910	10	755	10	721	11	3879
Unemployed	2	146	2	148	2	178	9	676	8	539	5	1687
Total N		6847		6726		8030		7621		7398		36622

Distribution of age, educational level and employment status by different study periods.

* Year 1985 is missing.

** Household income variable was divided into equal size quintiles, not shown in the table.

Prevalence (%) of self-reported insomnia and stress.

### Statistical analyses

**Figure 1:**
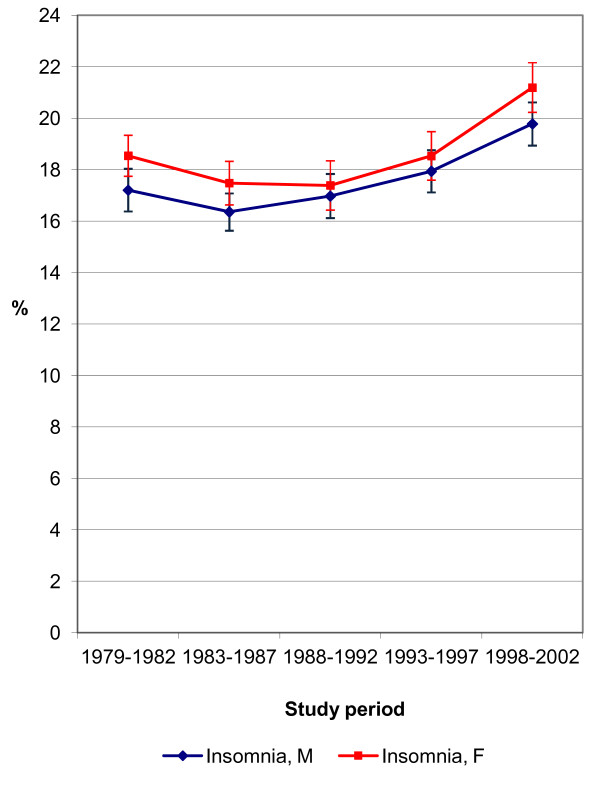
Age-standardised prevalence of self-reported insomnia 1979–2002 (%).

**Figure 2:**
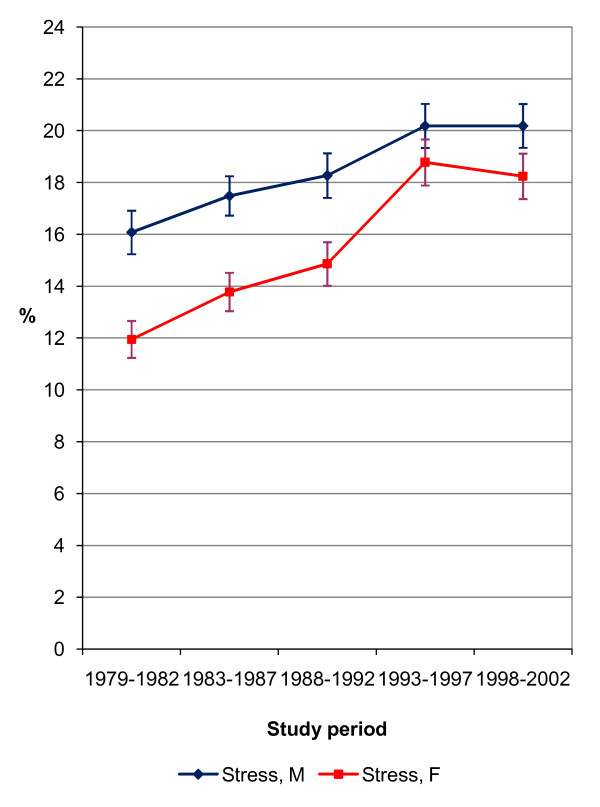
Age-standardised prevalence of self-reported stress 1979–2002 (%).

The prevalence of self-reported insomnia and stress was age-standardised using a direct standardisation method, where the total study population was applied as a standard population (Figures 1 and 2) [34]. We tested the statistical significance of the prevalence trend by means of logistic regression analysis. We performed the main analyses by logistic regression in order to evaluate the socio-economic differences in insomnia and stress between the years 1979–2002, and separated into five study periods (Tables 2,3,4,5). Age was used as a categorical variable (25–34, 35–44, 45–54, 55–64 years) in all the analyses. The results of the logistic regression analyses were presented as odds ratio (OR) estimates and their 95% confidence intervals (CI 95%). The highest educational level, employed respondents and the highest household income level were used as reference groups. The first logistic regression model explored the age-adjusted effects of each socio-economic variable for both total and separate study periods. With a view to exploring the changes in socio-economic differences in insomnia and stress over time, we tested and reported p-values for interaction effects between SES variables and the study period. We further conducted a logistic regression model for total study period, where all socio-economic variables were analysed simultaneously in order to see their independent adjusted effects. Finally, self-reported depression (measured by a single item ‘yes-no’ question) was included as a covariate in the last model in order to exclude the possible effect of depression on insomnia and stress.

**Table 2:** Age-adjusted odds ratios (95% Confidence Intervals) for self-reported insomnia by educational level, employment status and household income level during different study periods between years 1979–2002, men

	**Age-adjusted model**							**Age + all SES variables**		**Age + all SES variables + depression**	
	**79-82**	**83-87**	**88-92**	**93-97**	**98-02**	**1979-2002**^**1**^		**1979-2002**^**1**^		**1979-2002**^**1**^	
	**OR**	**OR**	**OR**	**OR**	**OR**	**OR**	**95% CI**	**OR**	**95% CI**	**OR**	**95% CI**
Educational level											
Highest	1.00	1.00	1.00	1.00	1.00	1.00		1.00		1.00	
Intermediate	1.07	1.07	1.11	1.15	**1.17**	**1.13**	**1.04-1.22**	**1.09**	**1.00-1.18**	1.07	0.98-1.17
Lowest	1.08	1.11	**1.24**	**1.26**	**1.27**	**1.20**	**1.11-1.29**	**1.11**	**1.02-1.20**	**1.10**	**1.01-1.20**
Interaction study period*educational level p = .964											
Employment status											
Employed	1.00	1.00	1.00	1.00	1.00	1.00		1.00		1.00	
Student	1.36	1.08	1.21	1.39	2.06	**1.44**	**1.17-1.77**	**1.43**	**1.16-1.76**	**1.35**	**1.08-1.70**
Retired	**2.74**	**2.54**	**2.09**	**2.06**	**1.72**	**2.18**	**1.99-2.39**	**2.16**	**1.96-2.38**	**1.73**	**1.56-1.92**
Unemployed	**2.47**	**3.06**	**3.01**	**2.09**	**2.51**	**2.48**	**2.22-2.76**	**2.42**	**2.16-2.71**	**1.76**	**1.55-2.00**
Interaction study period*employment status p = .009											
Household income											
Highest	1.00	1.00	1.00	1.00	1.00	1.00		1.00		1.00	
2. highest	0.99	1.04	**0.76**	0.96	1.03	0.95	0.87-1.04	**0.89**	**0.81-0.98**	**0.87**	**0.79-0.96**
Middle	1.02	1.12	0.86	0.95	0.93	0.96	0.88-1.06	**0.85**	**0.77-0.94**	**0.80**	**0.72-0.89**
2. lowest	1.02	0.98	1.03	0.89	0.98	0.98	0.90-1.08	**0.81**	**0.73-0.89**	**0.75**	**0.68-0.83**
Lowest	**1.30**	**1.39**	1.13	**1.35**	**1.55**	**1.34**	**1.23-1.46**	0.98	0.89-1.08	**0.89**	**0.80-0.99**
Interaction study period*household income level p = .269											

^1^Adjusted for study period.

*Significant odds ratios in bold.

**Table 3:** Age-adjusted odds ratios (95% Confidence Intervals) for self-reported insomnia by educational level, employment status and household income level during different study periods between years 1979–2002, women

	**Age-adjusted model**							**Age + all SES variables**		**Age + all SES variables + depression**	
	**79-82**	**83-87**	**88-92**	**93-97**	**98-02**	***1979-2002***^***1***^		***1979-2002***^***1***^		***1979-2002***^***1***^	
	**OR**	**OR**	**OR**	**OR**	**OR**	**OR**	**95% CI**	**OR**	**95% CI**	**OR**	**95% CI**
Educational level											
Highest	1.00	1.00	1.00	1.00	1.00	1.00		1.00		1.00	
Intermediate	0.98	0.95	1.04	1.05	1.04	1.00	0.93-1.08	0.98	0.91-1.06	0.98	0.91-1.06
Lowest	1.08	1.15	**1.20**	1.09	1.09	**1.12**	**1.04-1.21**	1.06	0.98-1.15	1.05	0.97-1.14
Interaction study period*educational level p = .054											
Employment status											
Employed	1.00	1.00	1.00	1.00	1.00	1.00		1.00		1.00	
Student	1.20	0.97	0.81	1.20	1.33	1.18	0.98-1.43	1.18	0.98-1.43	1.13	0.92-1.39
Housewife	1.04	1.08	1.05	0.97	**0.75**	1.01	0.92-1.11	0.99	0.90-1.10	0.95	0.86-1.06
Retired	**2.01**	**1.65**	**1.71**	**1.71**	**1.53**	**1.70**	**1.56-1.86**	**1.66**	**1.51-1.82**	**1.39**	**1.26-1.53**
Unemployed	**1.79**	**1.95**	**2.12**	**1.62**	**1.91**	**1.80**	**1.60-2.02**	**1.74**	**1.55-1.96**	**1.39**	**1.22-1.58**
Interaction study period*employment status p = .001											
Household income											
Highest	1.00	1.00	1.00	1.00	1.00	1.00		1.00		1.00	
2. highest	1.00	1.09	0.91	0.96	0.86	0.96	0.88-1.04	0.93	0.85-1.02	**0.91**	**0.83-0.99**
Middle	0.94	0.92	0.91	**0.83**	0.94	0.91	0.84-0.99	**0.85**	**0.78-0.93**	**0.84**	**0.76-0.92**
2. lowest	1.06	1.11	0.99	0.98	1.01	1.03	0.95-1.12	0.93	0.85-1.02	**0.90**	**0.81-0.99**
Lowest	**1.29**	1.14	1.17	1.06	1.05	**1.17**	**1.08-1.27**	0.99	0.90-1.08	**0.90**	**0.82-0.99**
Interaction study period*household income level p = .844											

^1^Adjusted for study period.

*Significant odds ratios in bold.

**Table 4:** Age-adjusted odds ratios (95% Confidence Intervals) for self-reported stress by educational level, employment status and household income level during different study periods between years 1979–2002, men

	**Age-adjusted model**							**Age + all SES variables**		**Age + all SES variables + depression**	
	**79-82**	**83-87**	**88-92**	**93-97**	**98-02**	***1979-2002***^***1***^		***1979-2002***^***1***^		***1979-2002***^***1***^	
	**OR**	**OR**	**OR**	**OR**	**OR**	**OR**	**95% CI**	**OR**	**95% CI**	**OR**	**95% CI**
Educational level											
Highest	1.00	1.00	1.00	1.00	1.00	1.00		1.00		1.00	
Intermediate	**0.71**	**0.68**	**0.73**	**0.81**	**0.80**	**0.76**	**0.71-0.82**	**0.73**	**0.68-0.79**	**0.67**	**0.62-0.73**
Lowest	**0.71**	**0.62**	**0.72**	0.88	**0.74**	**0.75**	**0.70-0.80**	**0.69**	**0.64-0.75**	**0.63**	**0.58-0.69**
Interaction study period*educational level p = .419											
Employment status											
Employed	1.00	1.00	1.00	1.00	1.00	1.00		1.00		1.00	
Student	1.30	0.75	1.05	**1.47**	0.76	**1.24**	**1.01-1.52**	1.15	0.93-1.41	1.05	0.83-1.31
Retired	**1.80**	**1.46**	1.06	0.95	**1.23**	**1.52**	**1.37-1.68**	**1.56**	**1.40-1.73**	1.11	0.99-1.25
Unemployed	**1.87**	**1.82**	**1.93**	**1.26**	**1.79**	**1.68**	**1.50-1.87**	**1.65**	**1.46-1.86**	1.06	0.92-1.21
Interaction study period*employment status p < .001											
Household income											
Highest	1.00	1.00	1.00	1.00	1.00	1.00		1.00		1.00	
2. highest	**0.80**	**0.74**	**0.81**	0.90	**0.79**	**0.81**	**0.74-0.89**	**0.86**	**0.78-0.94**	**0.82**	**0.75-0.91**
Middle	0.83	**0.78**	**0.65**	1.09	**0.72**	**0.81**	**0.74-0.88**	**0.86**	**0.78-0.94**	**0.80**	**0.73-0.89**
2. lowest	0.82	**0.67**	**0.72**	0.93	**0.74**	**0.79**	**0.72-0.86**	**0.81**	**0.74-0.89**	**0.76**	**0.68-0.84**
Lowest	1.05	0.93	0.91	**1.37**	**1.23**	**1.12**	**1.03-1.21**	**1.13**	**1.03-1.24**	1.04	0.94-1.16
Interaction study period*household income level p = .017											

^1^Adjusted for study period.

*Significant odds ratios in bold.

**Table 5:** Age-adjusted odds ratios (95% Confidence Intervals) for self-reported stress by educational level, employment status and household income level during different study periods between years 1979–2002, women

	**Age-adjusted model**							**Age + all SES variables**		**Age + all SES variables + depression**	
	**79-82**	**83-87**	**88-92**	**93-97**	**98-02**	***1979-2002***^***1***^		***1979-2002***^***1***^		***1979-2002***^***1***^	
	**OR**	**OR**	**OR**	**OR**	**OR**	**OR**	**95% CI**	**OR**	**95% CI**	**OR**	**95% CI**
Educational level											
Highest	1.00	1.00	1.00	1.00	1.00	1.00		1.00		1.00	
Intermediate	0.88	**0.68**	**0.67**	**0.87**	0.89	**0.79**	**0.74-0.85**	**0.76**	**0.71-0.82**	**0.72**	**0.67-0.79**
Lowest	1.03	**0.73**	**0.60**	**0.75**	0.95	**0.80**	**0.74-0.86**	**0.76**	**0.70-0.82**	**0.70**	**0.64-0.77**
Interaction study period*educational level p < .001											
Employment status											
Employed	1.00	1.00	1.00	1.00	1.00	1.00		1.00		1.00	
Student	**2.36**	1.33	**1.90**	1.12	1.28	**1.57**	**1.33-1.85**	**1.45**	**1.22-1.71**	**1.40**	**1.20-1.74**
Housewife	1.04	0.71	**0.67**	**0.71**	**0.69**	**0.79**	**0.71-0.88**	**0.75**	**0.67-0.84**	**0.69**	**0.61-0.78**
Retired	**2.21**	**1.47**	1.00	0.88	0.98	**1.36**	**1.22-1.52**	**1.29**	**1.16-1.45**	0.95	0.84-1.07
Unemployed	**2.39**	1.37	**1.61**	1.06	**1.56**	**1.45**	**1.28-1.64**	**1.37**	**1.20-1.55**	0.99	0.86-1.14
Interaction study period*employment status p < .001											
Household income											
Highest	1.00	1.00	1.00	1.00	1.00	1.00		1.00		1.00	
2. highest	0.88	1.01	**0.78**	0.89	**0.78**	**0.86**	**0.79-0.94**	**0.90**	**0.83-0.99**	**0.87**	**0.79-0.96**
Middle	1.04	**0.71**	**0.71**	**0.79**	**0.82**	**0.81**	**0.74-0.89**	**0.86**	**0.78-0.94**	**0.84**	**0.76-0.93**
2. lowest	1.08	0.96	**0.70**	0.87	0.86	**0.89**	**0.81-0.97**	0.95	0.86-1.04	0.91	0.82-1.01
Lowest	**1.51**	1.08	0.94	1.19	1.09	**1.18**	**1.08-1.29**	**1.26**	**1.14-1.39**	**1.18**	**1.06-1.31**
Interaction study period*household income level p = .040											

^1^Adjusted for study period.

*Significant odds ratios in bold.

Moreover, additional logistic regression analyses for extremely high stress category (2.5%) by socio-economic indicators were conducted in order to examine the robustness of the stress outcome (see Additional file 1). We carried out all statistical analyses separately for men and women using SPSS 17 for Windows (SPSS Corporation 2008).

## Results

### The prevalence of self-reported insomnia and stress

The overall prevalence of insomnia was 17.6% for men and 18.6% for women; the numbers for stress were 18.5% for men and 15.6% for women. The trend in the prevalence of insomnia was slightly u-shaped; a decreasing trend was seen after the study period 1979–1982, but then the prevalence began to increase again in the latest study periods (Figure 1). During the last study period, 1998–2002, there was a statistically significant (p < .001) almost three percentage points increase in insomnia, among both men and women compared to the first study period. As for stress prevalence, there was linear increase until study period 1993–1997 among both sexes, indicating statistically significant (p < .001) four to seven percentage points increase compared to the first study period (Figure 2). For the last study period, 1998–2002, the prevalence of stress remained similar to the 1993–1997 study period.

### Socio-economic differences in self-reported insomnia

In the age-adjusted model, during the total study period 1979–2002 (Tables 2 and 3), the lowest educated had more insomnia compared to the highest educated among both men (OR 1.20, 95% CI 1.11-1.29) and women (OR 1.12, 95% CI 1.04-1.21). Retired men (OR 2.18, 95% CI 1.99-2.39) and women (OR 1.70, 95% CI 1.56-1.86) and unemployed men (OR 2.48, 95% CI 2.22-2.76) and women (OR 1.80, 95% CI 1.60-2.02) had more insomnia compared to the employed. In terms of the household income level, those in the lowest income level had more insomnia compared to the highest income level among men (OR 1.34, 95% CI 1.23-1.46) and women (OR 1.17, 95% CI 1.08-1.27). After simultaneous adjustment for all SES variables, employment status differences remained similar to the age-adjusted model among both sexes. However, the educational level differences were no longer statistically significant among women. Furthermore, the lowest household income level no longer differed from the highest income level among women, and among men, the association of insomnia with household income level turned u-shaped as the intermediate levels of income had the lowest insomnia. Following further adjustment for self-reported depression; intermediate level of education no longer differed from the highest education among men; and compared to the highest income, respondents on all the other levels of income had less insomnia among both men and women.

For insomnia, there was a statistically significant interaction between study period and employment status among men (p = 0.009) and women (p = 0.001) (Tables 2 and 3). During the recession and period of high unemployment in 1993–1997, differences in insomnia narrowed between the employed and unemployed respondents among both sexes due to the decrease in insomnia among the unemployed. No significant changes existed in insomnia by educational level or household income level over different study periods.

### Socio-economic differences in self-reported stress

In the age-adjusted model for the total study period (Tables 4 and 5), the lowest educated experienced less stress compared to the highest educated among both men (OR 0.75, 95% CI 0.70-0.80) and women (OR 0.80, 95% CI 0.74-0.86). In addition, less stress was observed with intermediate education. Those associations remained statistically significant even after mutual adjustment for other SES variables. In the case of employment status, retired men (OR 1.52, 95% CI 1.37-1.68) and women (OR 1.36, 95% CI 1.22-1.52), and unemployed men (OR 1.68, 95% CI 1.50-1.87) and women (OR 1.45, 95% CI 1.28-1.64) had more stress compared to the employed in the age-adjusted model, and following adjustment for other SES variables. Moreover, students reported higher stress and housewives less stress. Regarding household income, compared to the highest level of income, stress was less common among the intermediate levels of income, but more common among those in the lowest household income level among both men (OR 1.12, 95% CI 1.03-1.21) and women (OR 1.18, 95% CI 1.08-1.29) even after adjustment for educational level and employment status.

After further adjustment for self-reported depression as a covariate in the models, statistically significant associations in stress vanished for the retired and unemployed respondents among both men and women, and for the lowest income level among men.

Complementary analysis with extremely high stress-category ( ‘my life is nearly unbearable’) as an outcome measure resulted in reversed educational differences compared to the original wider stress classification; in the age-adjusted model lower educated had more stress compared to highest educated among both men and women (see Additional file 1). However, after mutual adjustment for other SES variables, educational level differences were no longer statistically significant. Employment status gradients resembled association of wider stress, as well as the lowest level of household income was associated with more stress according to both stress classifications.

In men (Table 4), a statistically significant change over time appeared in stress by employment status (p < 0.001) and household income (p = 0.017). In particular, there were narrowing differences during the period of recession in 1993–1997, however, at the same time some associations increased; statistically significantly higher odds for stress were among students and those in the lowest level of income. In women (Table 5), change over time was statistically significant for stress by all socio-economic indicators. In women narrowing of the differences by employment status (p < 0.001) during recession were even more pronounced than in men; students, early retired and unemployed had no longer statistically significantly more stress compared to the employed. Educational level differences had a statistically significant change over time (p < 0.001); no differences existed during the first and last study periods whereas the other study periods showed clear reversed educational differences in stress. Changes in household income level (p < 0.040) differences in stress fluctuated over time with no pronounced pattern.

## Discussion

Our aim was to study the prevalence and the socio-economic differences in self-reported insomnia and stress over the years 1979–2002 in Finland. Compared to the first study period, 1978–1982, there was increase in the prevalence of stress until the period 1993–1997 among men and women. There was also an increase in the prevalence of self-reported insomnia among both men and women during the last study period, 1998–2002. Consistently more insomnia and stress was among the unemployed and retired (early retirees in this data). Lowest education was associated with more insomnia especially among men, and less stress among both sexes. Those in the intermediate levels of household income had least stress. Income level differences in insomnia were less consistent. Socio-economic differences slightly fluctuated over the total period 1979–2002; however, there were no substantial changes in socio-economic differences in insomnia and stress.

This study provided new information about trends in self-reported insomnia and stress by socio-economic factors utilising a repeated cross-sectional study design on a 24-year time scale. It benefited from the use of nationally representative population survey data, which were supplemented with reliable educational level and household income data from Statistics Finland. As a limitation of the survey, the data included only non-specific single-item measures of insomnia and stress, which may cover a wide range of psychological symptoms from transient to severe, chronic symptoms. We have pointed out in this article that these measures are not used for any diagnostic purposes but instead to represent the subjective dimension of mental well-being which can be easily used to monitor differences in population subgroups [7]. However, as was demonstrated in our prior study, single-item measures of insomnia and extremely high stress had significant associations with cause-specific mortality, such as coronary heart disease mortality and so-called unnatural mortality (including alcohol-related mortality, accidents, violence and suicide) [13].

Some other methodological issues need to be addressed concerning the variables used. In this study we used ‘more stress than in people general’ and ‘my life is nearly unbearable’ categories combined as indicator for stress. Educational differences were reversed in stress; those in the highest education were more stressed compared to the less educated. This finding is in line with many other previous research, however, the phenomenon is not clearly explained in the literature [24,35]. It has been proposed, especially related to work stress, that those with higher education have for example gained occupational position with greater responsibilities, higher expectations and higher stress [25]. One explanation is relating to the complexity of the stress construct and measurement. In general, our stress measure is exploring only perception of stress, with no further information about sources of stress, or possible coping resources or outcomes. In additional analyses we used ‘my life is nearly unbearable’ category alone as an indicator for extremely high stress (2.5% in the exposed group). Extremely high stress was more common in the lower levels of education compared to the highest education, although the associations were not independent of other socio-economic factors. Our results indicate that even though stress seems to be more common among the highest educated, some of the most extreme stressful situations may be experienced among those in the lowest education. Specific measures of stress would be needed in order to examine different sources and exposures of stress, duration, as well as coping resources and responses. Stress, and less consistently insomnia, also produced an u-shaped distribution with household income level, suggesting intermediate levels being protective for insomnia and stress. More stress and insomnia in the lowest income group may relate, for example, to low social participation and material resources, whereas more symptoms among the highest income group might relate to the factors associated with the higher social position and occupational status, as was discussed with education.

Psychological symptoms are known to be associated with each other with complex interrelations. In our study, Pearson’s correlations (p < 0.001) between insomnia and depression were r = 0.37 in males and r = 0.34 in females; and between stress and depression r = 0.40 in males and r = 0.38 in females. We made adjustment for self-reported depression in order to control the possible effect of depression on the associations for insomnia and stress. Most significant effect on following this adjustment was for retired and unemployed respondents, which had no longer statistically significantly higher stress among neither of sexes. Therefore, some of the employment status differences in stress may be explained by depression. However, if stress and insomnia symptoms are preceding and predictors of depression, as some of the evidence is demonstrating [36-38], then adding depression in the analyses may have caused over adjustment in the models.

Moreover, insomnia and stress are commonly thought to relate to each other [14]; for example, work stress is linked to insomnia [39-41]. In our data, 39% of those having stress reported insomnia, and 41% of those who reported insomnia also had stress; however, the cross-sectional study design of our data does not allow us to make conclusions about the causality of the stress-insomnia relationship, or any other studied associations. Trends in the prevalence of insomnia and stress were not identical to either each other or to what we previously found using the same data with self-reported depression, which for example showed a decreasing trend contrary to the increasing trend in insomnia and stress [6]. Even though insomnia and stress are known to be related to each other and other indicators for mental health problems, they may also produce an independent risk factor for health and well-being.

Studies of the effects of retirement on mental health, including sleep outcomes, has produced inconsistent findings showing both improvement and increase in symptoms [42,43]. In a longitudinal follow-up study, sleep disturbances have been found to improve after retirement, which were explained by removal of work-related risk factor exposures. Retirement on health grounds was, however, associated with increase in sleep disturbances following retirement [44]. In the Finnish register-based follow-up study sleeping problems were found to be associated with subsequent disability retirement [10]. In our study retired respondents, which were early retirees, had more insomnia and stress. Early retirement is known to be associated with lower mental and physical health [45-47], and mental and musculoskeletal disorders are the most common reasons for granting disability pension in Finland [48-50].

Over the years, the overall response rate of the “Health Behaviour and Health among the Finnish Adult Population” survey has decreased from 84% down to 65%. Similar rates have been found in other population surveys [51]. We have conducted several non-respondent analyses on our survey data [52,53] in which we found that the non-respondents were more likely to be male, young and lower educated. Gender and educational differences in the response rate have further widened over time. Additionally, we found higher total and cause-specific excess mortality (such as due to alcohol, external causes, suicide) among survey non-respondents, which was partly explained by educational and income differences between respondents and non-respondents. The results of those non-respondent analyses indicate that the non-respondents may have, for example, unhealthy lifestyles, more severe illnesses and mental health problems, and may also differ from the respondents in terms of self-reported psychological symptoms. In the present study, respondents with missing data on insomnia and stress variables were also more likely to be in the lower SES groups.

After the economic boom of the 80s, Finland experienced its most severe financial crisis to date at the beginning of the 90s. The economic recession caused unemployment and loss of income for a wide population regardless of socio-economic background. Several studies have examined the implications of the recessionary period for psychological health, which is hypothesised to have deteriorated during that time [54-56]. This was only partially supported in a Finnish longitudinal cohort study, which showed no drastic decline in sleep quality during 1991–1995 except among the prospectively unemployed, most of whom were blue-collar workers [57]. We found no statistically significant increase in insomnia during the period of high unemployment in 1993–1997. The prevalence of stress had a linearly increasing trend, which during recession period strengthened especially among women. However, general increase in stress was not attributable to any specific socio-economic levels. During the recession, changes in socio-economic differences were somewhat inconsistent, but differences especially between the employed and unemployed respondents rather narrowed in both insomnia and stress. This indicates that when national unemployment rates were relatively high, being unemployed was obviously not as selective as usual. Valkonen et al. [58] found that economic recession slowed down rather than sped up the growth of relative inequalities in mental health related mortality (such as alcohol-related causes, accidents and suicide) in Finland.

The prevalence of self-reported insomnia increased over the last time period, 1998–2002. Various studies have found similar evidence for an increase in insomnia. In a review and re-analysis conducted in Finland it was found that insomnia-related symptoms increased during 1995–2005 among the general population and especially among the employed working-aged population [28]. It was concluded that the increase in insomnia-related symptoms may be due to changes in working life that have raised the employees’ stress level. In the Swedish population study of women [29] the proportion of respondents with sleeping problems almost doubled from 1968 to 2004. Perceived poor economic status, poor family and social situation as well as mental stress were related to sleeping problems in women [29]. In our data the increase in insomnia was evenly distributed (excluding housewives) over different socio-economic groups, making it difficult to account for socio-economic inequality.

Although socio-economic differences slightly fluctuated over the total period 1979–2002, significant changes in socio-economic differences were rare. It is noteworthy, that some of the differences in insomnia and stress were reversed and curvilinear. Future studies are needed to explore the complexity and significance of socio-economic differences, especially in stress, as well as the growing prevalence of psychological symptoms.

## Conclusions

Insomnia and stress have become more prevalent over the years. The socio-economic differences in self-reported insomnia and stress fluctuated but did not change substantially during the total study period 1979–2002. Some of the socio-economic gradients in stress and insomnia were curvilinear, and reversed depending on the measure and classification used.

## Competing interests

The authors declare that they have no competing interests.

## Authors’ contributions

KTM processed the data, carried out the statistical analyses, drafted and finalised the manuscript. TPM and AHH supervised the first author and participated in interpreting the data and drafting the manuscript. TTH participated in interpreting the data and provided advice on the statistical analyses. RSP took part in the coordination of the study, supervised the first author and was involved in interpreting the data and drafting the manuscript. All authors revised the text critically for important intellectual content and read and approved the final manuscript.

## Pre-publication history

The pre-publication history for this paper can be accessed here:

http://www.biomedcentral.com/1471-2458/12/650/prepub

## Supplementary Material

Additional file 1 **Table S1.** Odds ratios (95% Confidence Intervals) for extremely high stress (‘my life is nearly unbearable’) by educational level, employment status and household income level for total study period 1979–2002. Men and women. Click here for file
