# Supplementary material for: Socio-economic differences in self-reported insomnia and stress in Finland from 1979 to 2002: a population-based repeated cross-sectional survey
Source: BMC Public Health. 2012 Aug 13;12:650. doi: 10.1186/1471-2458-12-650 (PMC3509034; doi:10.1186/1471-2458-12-650)
Supplement: Additional file 1 — Table S1. Odds ratios (95% Confidence Intervals) for extremely high stress (‘my life is nearly unbearable’) by educational level, employment status and household income level for total study period 1979–2002. Men and women. [file 1471-2458-12-650-S1.doc]

Additional table 1. Odds ratios (95% Confidence Intervals) for extremely high stress (‘my life is nearly unbearable’) by educational level, employment status and household income level for total study period 1979-2002. Men and women.

|  | Men, 1979-2002 | | Women, 1979-2002 | |
| --- | --- | --- | --- | --- |
|  | Age-adjusted^1^ | Age+  all SES variables adjusted^1^ | Age-adjusted^1^ | Age+  all SES variables adjusted^1^ |
| Educational  level | OR (95% CI) | OR (95% CI) | OR (95% CI) | OR (95% CI) |
| Highest | 1.00 | 1.00 | 1.00 | 1.00 |
| Intermediate | **1.37 (1.13-1.66)** | 1.16 (0.95-1.43) | **1.24 (1.03-1.49)** | 1.00 (0.82-1.21) |
| Lowest | **1.54 (1.27-1.86)** | 1.20 (0.97-1.48) | **1.54 (1.28-1.87)** | 1.18 (0.96-1.44) |
|  |  |  |  |  |
| Employment  status |  |  |  |  |
| Employed | 1.00 | 1.00 | 1.00 | 1.00 |
| Student | **2.18 (1.40-3.39)** | **1.99 (1.27-3.11)** | 1.53 (0.99-2.34) | 1.17 (0.74-1.82) |
| Housewife | - | - | 1.09 (0.85-1.41) | 0.87 (0.67-1.13) |
| Retired | **3.16 (2.56-3.92)** | **2.57 (2.04-3.23)** | **2.90 (2.33-3.61)** | **2.15 (1.71-2.71)** |
| Unemployed | **4.55 (3.73-5.56)** | **3.66 (2.94-4.55)** | **2.91 (2.31-3.68)** | **2.12 (1.65-2.71)** |
|  |  |  |  |  |
| Household  income |  |  |  |  |
| Highest | 1.00 | 1.00 | 1.00 | 1.00 |
| 2. highest | 0.82 (0.64-1.06) | **0.74 (0.57-0.96)** | 1.21 (0.95-1.56) | 1.14 (0.89-1.47) |
| Middle | 1.02 (0.81-1.30) | 0.81 (0.63-1.05) | 1.24 (0.96-1.59) | 1.06 (0.82-1.37) |
| 2. lowest | **1.30 (1.04-1.64)** | 0.94 (0.73-1.20) | **1.61 (1.27-2.04)** | **1.33 (1.04-1.71)** |
| Lowest | **2.19 (1.77-2.70)** | **1.31 (1.03-1.66)** | **2.86 (2.30-3.56)** | **2.17 (1.70-2.76)** |

^1^Adjusted for study period

*Significant odds ratios in bold
